# Mental Health Research During the COVID-19 Pandemic: Focuses and Trends

**DOI:** 10.3389/fpubh.2022.895121

**Published:** 2022-07-26

**Authors:** Yaodong Liang, Li Sun, Xin Tan

**Affiliations:** ^1^Law School, Changsha University, Changsha, China; ^2^Department of Psychology, University of Toronto St. George, Toronto, ON, Canada; ^3^Centre for Mental Health and Education, Central South University, Changsha, China

**Keywords:** COVID-19, mental health, bibliometric analysis, keyword clustering, focuses, trends

## Abstract

**Background:**

The COVID-19 pandemic has profoundly influenced the world. In wave after wave, many countries suffered from the pandemic, which caused social instability, hindered global growth, and harmed mental health. Although research has been published on various mental health issues during the pandemic, some profound effects on mental health are difficult to observe and study thoroughly in the short term. The impact of the pandemic on mental health is still at a nascent stage of research. Based on the existing literature, we used bibliometric tools to conduct an overall analysis of mental health research during the COVID-19 pandemic.

**Method:**

Researchers from universities, hospitals, communities, and medical institutions around the world used questionnaire surveys, telephone-based surveys, online surveys, cross-sectional surveys, systematic reviews and meta-analyses, and systematic umbrella reviews as their research methods. Papers from the three academic databases, Web of Science (WOS), ProQuest Academic Database (ProQuest), and China National Knowledge Infrastructure (CNKI), were included. Their previous research results were systematically collected, sorted, and translated and CiteSpace 5.1 and VOSviewers 1.6.13 were used to conduct a bibliometric analysis of them.

**Result:**

Authors with papers in this field are generally from the USA, the People's Republic of China, the UK, South Korea, Singapore, and Australia. Huazhong University of Science and Technology, Hong Kong Polytechnic University, and Shanghai Jiao Tong University are the top three institutions in terms of the production of research papers on the subject. The University of Toronto, Columbia University, and the University of Melbourne played an important role in the research of mental health problems during the COVID-19 pandemic. The numbers of related research papers in the USA and China are significantly larger than those in the other countries, while co-occurrence centrality indexes in Germany, Italy, England, and Canada may be higher.

**Conclusion:**

We found that the most mentioned keywords in the study of mental health research during the COVID-19 pandemic can be divided into three categories: keywords that represent specific groups of people, that describe influences and symptoms, and that are related to public health policies. The most-cited issues were about medical staff, isolation, psychological symptoms, telehealth, social media, and loneliness. Protection of the youth and health workers and telemedicine research are expected to gain importance in the future.

## Introduction

Although the impacts of the COVID-19 pandemic will be recorded in human medical history and in socio-economic history, various psychological consequences regarding mental health among populations cannot be ignored, including stress, anxiety, depression, frustration, insomnia, and so on. Researchers from universities, hospitals, communities, and medical institutions worldwide have been focusing on mental health problems during the pandemic. They have used questionnaire surveys, telephone-based surveys, online surveys, cross-sectional surveys, systematic reviews and meta-analysis, and systematic umbrella reviews to investigate mental health problems during the pandemic. Two years after the outbreak of the COVID-19, the pandemic has gradually subsided in some countries, while others have adopted a strategy of coexisting with the virus. If more deadly mutant strains do not appear in the future, it is very likely that the pandemic will not climax again. It is pertinent to summarize and study mental health research during the pandemic, because many psychological problems have arisen as a result, and there has been significant interest in research on such issues in the previous two years.

As an effective quantitative analysis method, bibliometrics can be used not only to assess the quality and quantity of published papers, but also to explore research focuses and trends, the distribution of authors and institutions, the impact of publications, journals, and different countries regarding research contributions to the theme. Due to the rapid growth in research in this area, there are now over 1,000 academic papers, and accordingly, it would appear necessary to investigate important, valid, and meaningful information from large databases to guide scientific research. The authors used CiteSpace and VOSviewers to determine the focuses and trends in this regard.

## Methods

### Data Analysis and Visualization

The authors searched the Web of Science (WOS), ProQuest Academic Database (ProQuest), and China National Knowledge Infrastructure (CNKI) to extract publications related to mental health and COVID-19. Their previous research results were systematically collected, sorted, and translated, and CiteSpace 5.1 and VOSviewers 1.6.13 were used to conduct a bibliometric analysis of them.

### Data Source and Search Strategy

Our team selected 1,226 papers from 2019 to 2022 using three combinations of keywords, mental health and COVID-19, mental health and new coronavirus, and mental health and novel coronavirus, from the three academic paper databases, WOS, ProQuest, and CNKI. Two explanations are necessary here, the first is about the keywords and the second is about the databases. (1) The reason we used new or novel coronavirus as keywords was that the name COVID-19 has not been determined about 2 years ago. In order not to miss relevant research results, we also included these synonyms as keywords for the search. (2) Among the three databases, WOS and ProQuest, in which most of the English-language papers were published, are well-known to scholars all around the world. However, the CNKI database is not as popular as WOS or ProQuest given that most of the papers in CNKI were published in Chinese. We chose to use the CNKI data for the following three reasons: first, China was the most affected country during the COVID-19 outbreak and Chinese academic journals published significant research on mental health. Second, CNKI is the largest Chinese academic database. Third, after the outbreak, the Chinese government's virus clearance policy has been implemented and continues to date. Strict control has helped suppress the spread of the virus, but has also likely had mental health implications, given the severe reduction in social interactions. Therefore, we think that the Chinese database is appropriate and useful in this study.

About 50% of the articles were from the WOS, about 10% of the articles from ProQuest, and about 40% from CNKI. Basic information such as title, author, institution, country, abstract, keywords, methods, results, and conclusions of all articles, if not in English, are translated into English and analyzed using SiteSpaceII and VOSviewers. Since the keywords include COVID-19 and mental health, synonyms such as novel coronavirus and psychological distress spontaneously appeared while searching. Words that are closely related to the subject, such as public health, quarantine, and insomnia, were most frequently mentioned.

## Results

**Figure 1:**
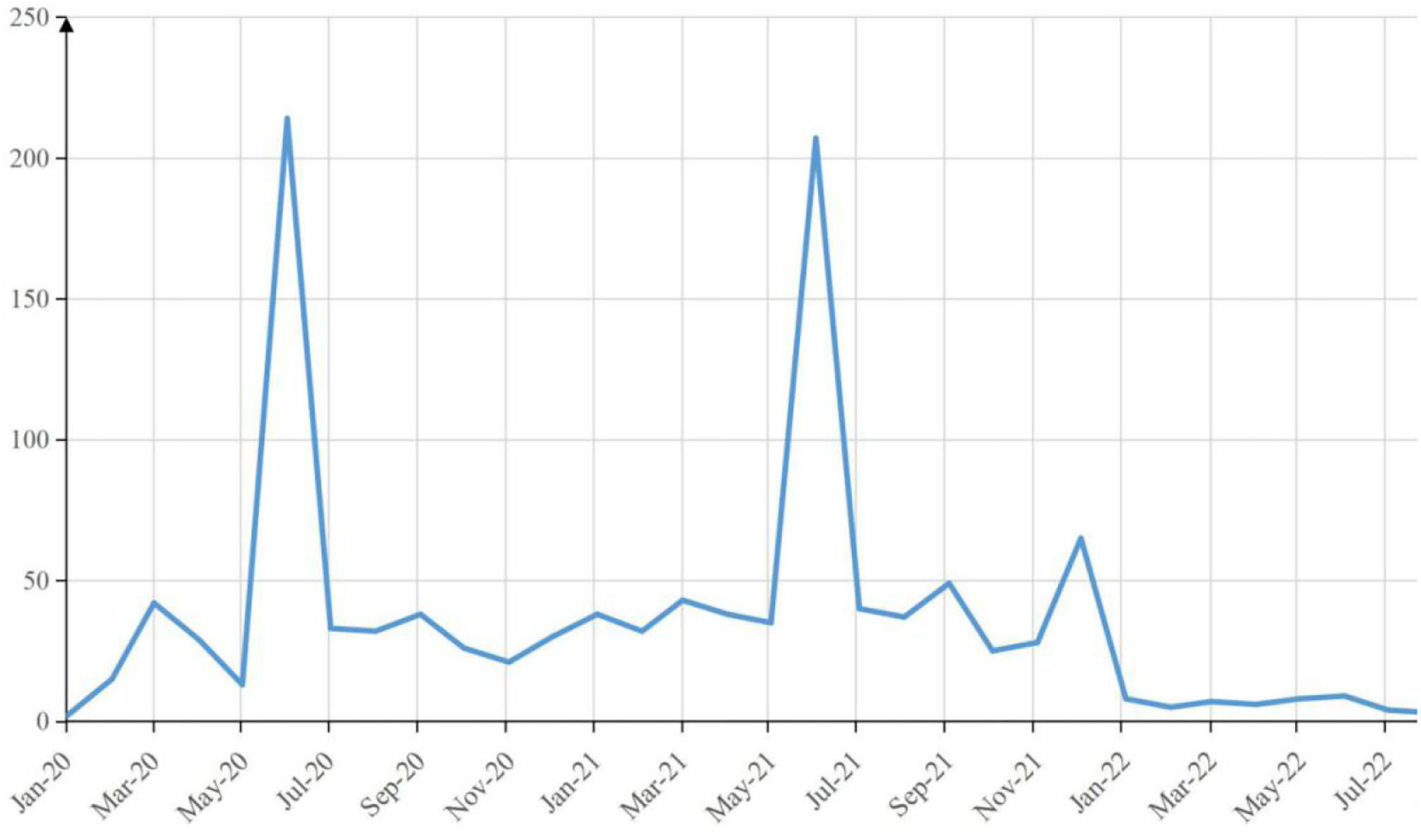
The volume of COVID-19 and mental health-related articles in 2020–2022.

Most articles were published during the period from February 2020 to July 2022, including those pre-published online from April to July, and only one article that had been published in 2019 was included. Judging from the line chart above, since the volume of COVID-19 and mental health-related articles had already risen two times in June 2020 and June 2021 and then remained low until now, it is high time to conclude a previous study on COVID-19 and mental health, to sort out the foci of those studies, and to analyze and predict future trends (Figure 1).

Scholars from around the world have contributed to the study of mental health issues during the COVID-19 pandemic. The top 10 countries with the largest quantum of publications related to mental health during COVID-19 are the USA, People's Republic of China, England, Canada, Australia, India, Italy, Japan, Iran, and Germany. Wide and active participation of several countries has laid a solid foundation for its future development. Universities, hospitals, communities, and medical institutions around the world have conducted sample surveys of patients, students, community residents, medical workers, and other sample populations of considerable sample sizes since the outbreak. Survey and research methods include questionnaire survey, telephone-based survey, online survey, cross-sectional survey, systematic review and meta-analyses, and systematic umbrella review (Table 1).

**Table 1:** Top 20 countries.

**Ranking**	**Frequency**	**Country**	**Ranking**	**Frequency**	**Country**
1	280	USA	11	27	Spain
2	223	China	12	26	Brazil
3	85	England	13	22	Saudi Arabia
4	69	Canada	14	19	Pakistan
5	68	Australia	15	18	Turkey
6	54	India	16	12	Bangladesh
7	50	Italy	17	11	Sweden
8	41	Japan	18	10	Singapore
9	37	Iran	18	10	Poland
10	27	Germany	20	9	Malaysia

**Figure 2:**
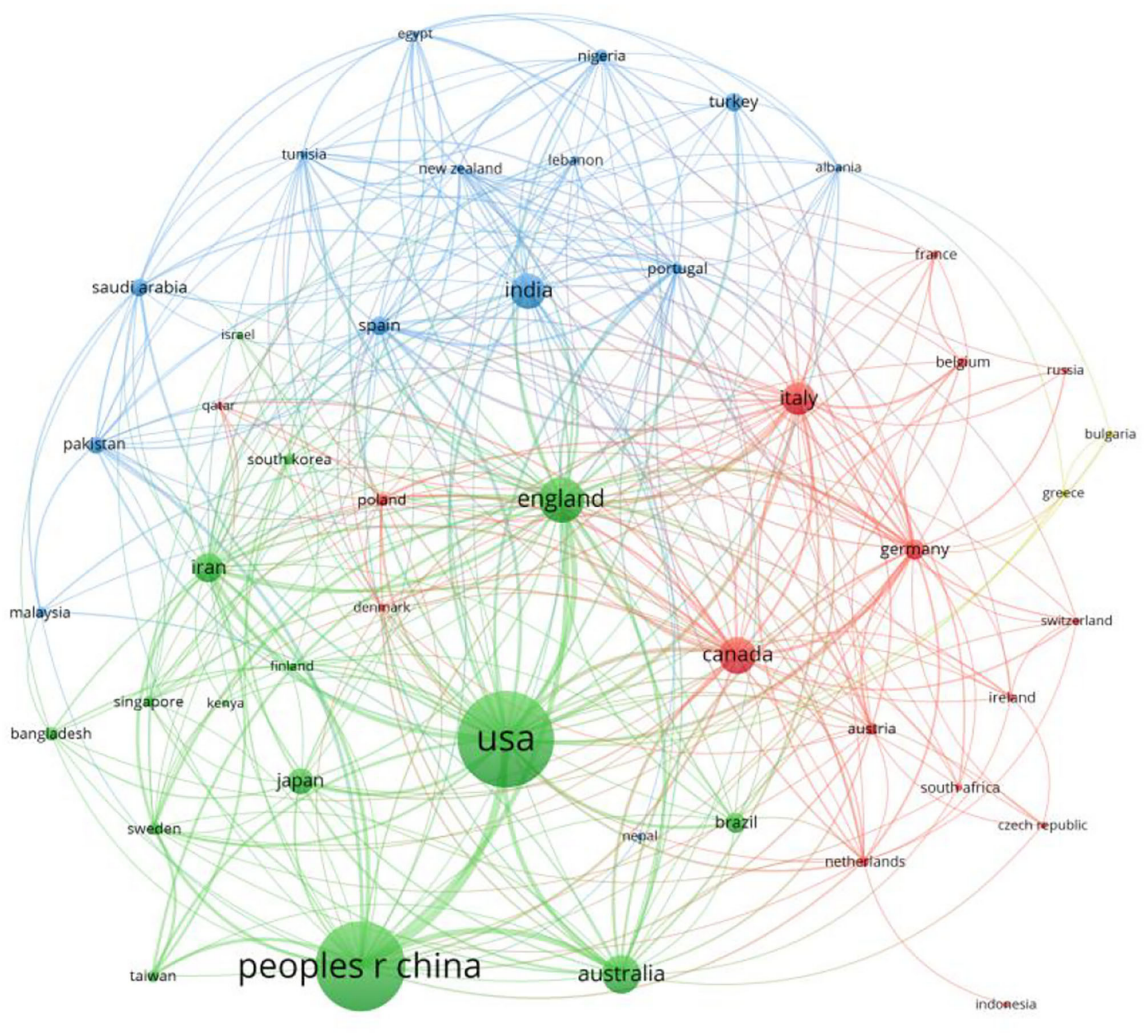
Country or region co-occurrence.

Most papers are from the USA, the People's Republic of China, England, Australia, Canada, India, Italy, Iran, Japan, and Germany. Judging from the country or region co-occurrence graph, England and Canada are in the center of this graph, with India, Poland, Denmark, Spain, South Korea, Portugal, Italy, and Canada around them. England, Australia, Canada, Japan, Brazil, India, Iran, and Germany have done significant research work in this field. In addition, the number of related research papers in the USA and China is significantly larger than that in all other countries (Figure 2).

In Table 2, we can see that most names of the top 20 authors are Asian names, and they are mainly from China. Six of them published more than 10 articles by the end of 2021. In the extended ranking, we find that the authors who have published a large number of papers are generally from the USA, China, the UK, South Korea, Singapore, and Australia. The authors Griffiths MD, Cheung T, Xiang Y, Lin C, Wang Y, and Zhang L were very active in this field of study.

**Table 2:** Top 20 authors.

**Ranking**	**Frequency**	**Author**	**Ranking**	**Frequency**	**Author**
1	14	Xiang YT	7	7	Zvolensky MJ
2	13	Zhang L	12	6	Ng CH
2	13	Wang Y	12	6	Pakpour AH
2	13	Cheung T	14	5	Li W
5	11	Li Y	14	5	Li X
5	11	Griffiths MD	14	5	Garey L
7	7	Li L	14	5	Zhong BL
7	7	Zhang Y	14	5	Wang W
7	7	Zhang Q	14	5	Yang Y
7	7	Lin CY	20	4	Hu SH

**Figure 3:**
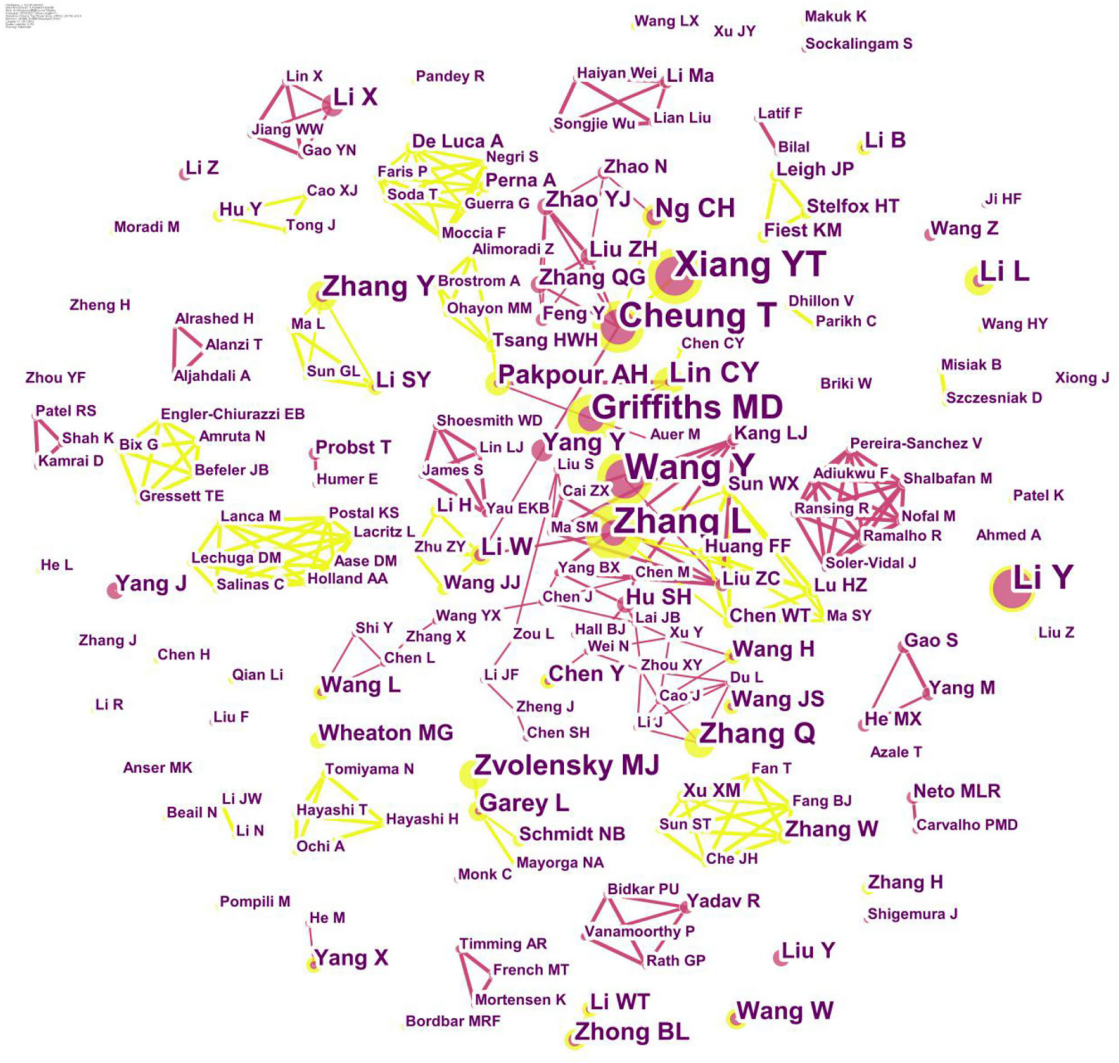
Author co-occurrence.

**Figure 4:**
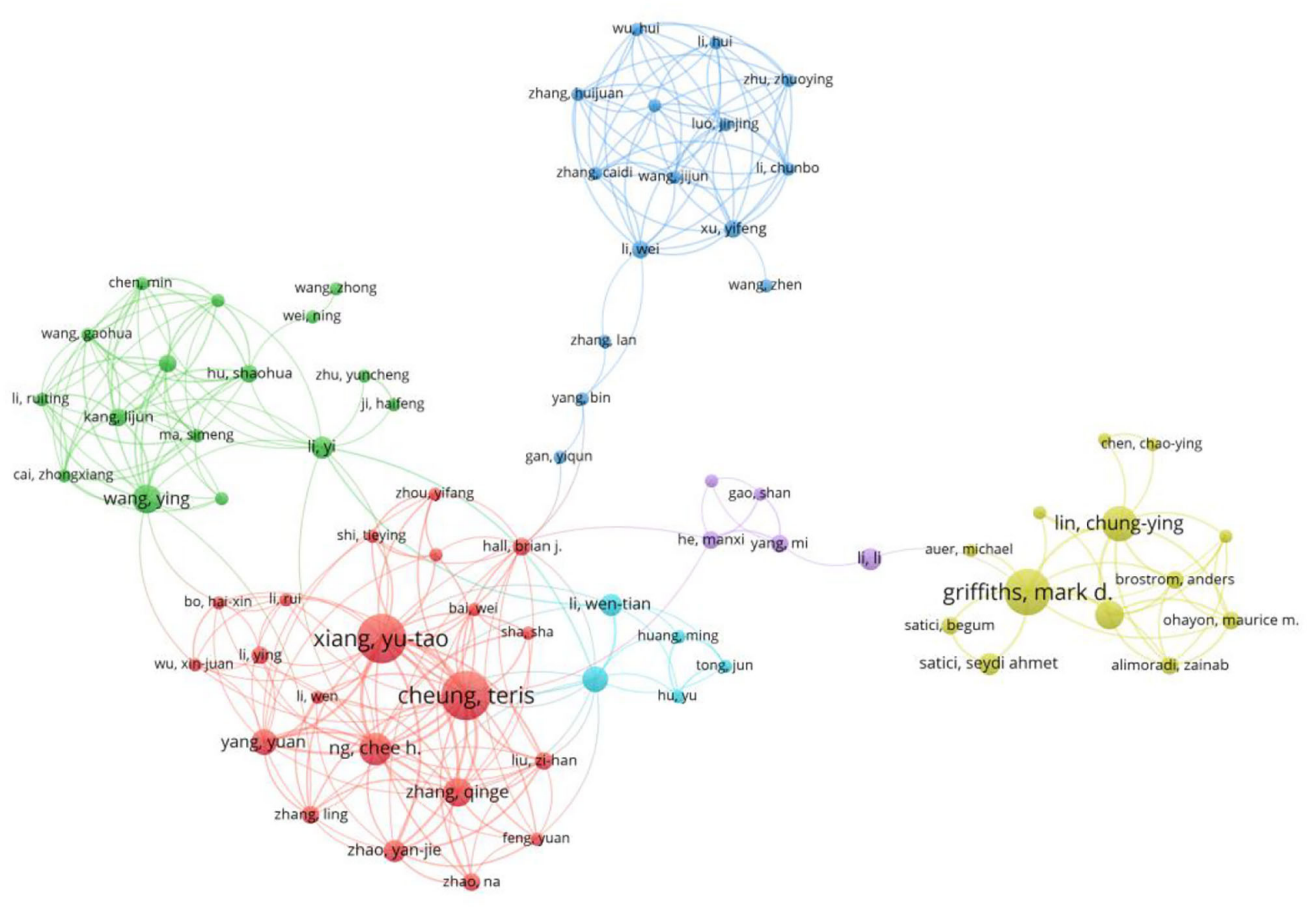
Author co-occurrence groups.

In the abovementioned graphs, we can see six groups of related authors. The VOSviewer was used to describe the partnership between them. Though six colors were used to separate these groups, there were still lines connecting the groups to represent the partnership between them. We can take Cheung T and Xiang Y as the center of the largest group. Another group with Griffiths MD and Lin C as its center was also significant (Figures 3, 4).

**Figure 5:**
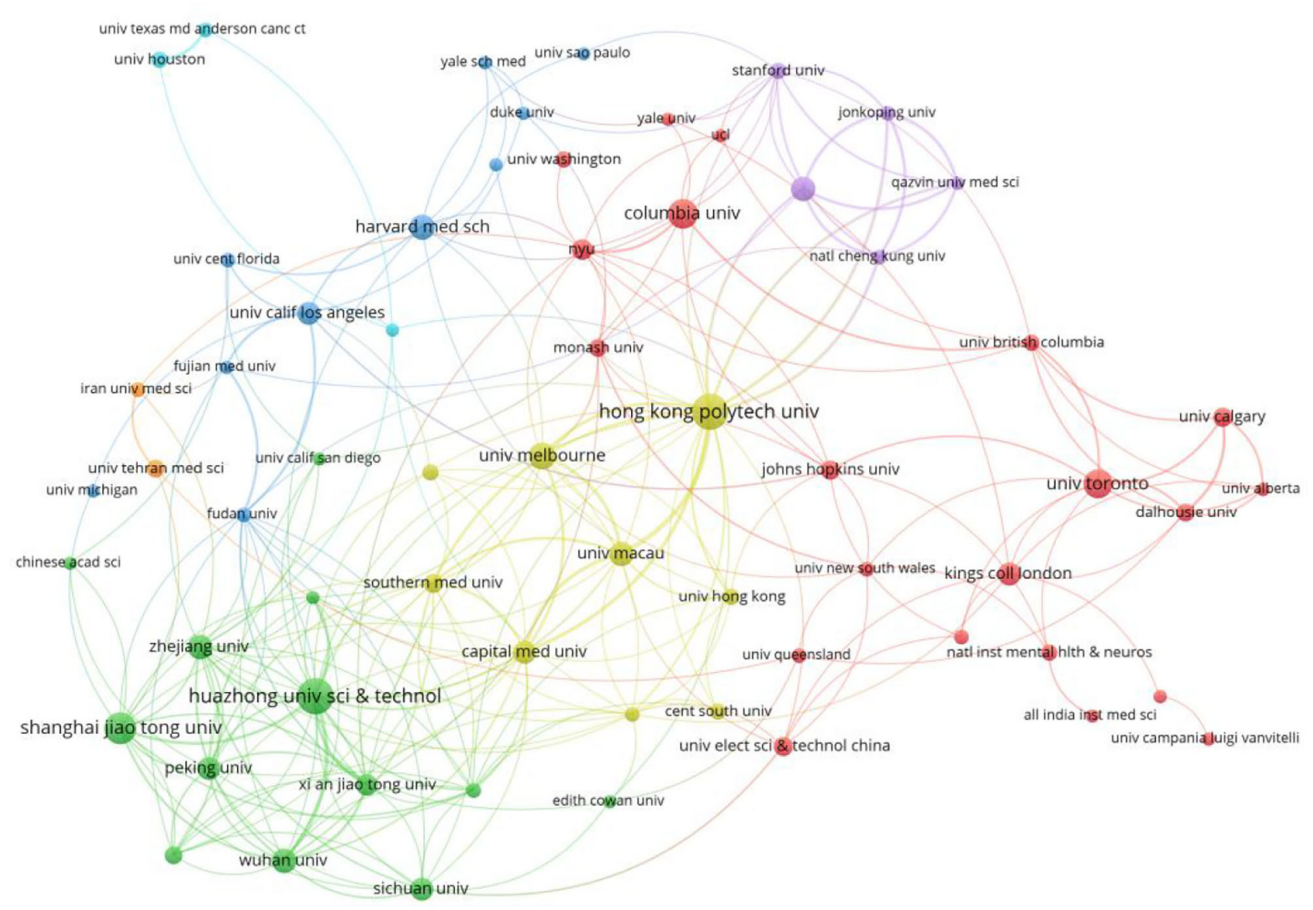
Institutions' co-occurrence.

The top five institutions are Huazhong University of Science and Technology, Hong Kong Polytechnic University, Shanghai Jiao Tong University, Columbia University, and the University of Toronto. Meanwhile, the top five institutions in centrality are the University of Macau, the University of Melbourne, Columbia University, Wuhan University, and the University of Toronto. It is worth mentioning that Huazhong University of Science and Technology and Wuhan University are located in the city of Wuhan, one of the areas most affected by the virus through the outbreak. The society and economy of the city temporarily stagnated at the time, and its medical system was once paralyzed. Eventually, Wuhan City's medical system was fully recovered. The University of Toronto, Columbia University, and the University of Melbourne have played an important role in the research of mental health problems during the COVID-19 pandemic (Table 3 and Figure 5).

**Table 3:** Top 20 institutions.

**Ranking**	**Frequency**	**Centrality**	**Institution**
1	25	0.18	Huazhong University of Science and Technology
2	25	0.14	Hong Kong Polytechnic University
3	21	0.12	Shanghai Jiao Tong University
4	19	0.56	Columbia University
5	18	0.44	The University of Toronto
6	16	0.61	The University of Melbourne
7	16	0.35	Harvard Medical School
8	14	0.78	The University of Macau
9	14	0.50	Wuhan University
10	13	0.12	Kings College London
11	13	0.01	Capital Medical University
12	12	0	Nottingham Trent University
13	11	0	Peking University
14	11	0.22	New York University
15	10	0.12	Zhejiang University
16	10	0	The University of California Los Angeles
16	10	0	Sichuan University
18	9	0.21	Dalhousie University
19	9	0	Xi An Jiao Tong University
20	8	0	The University of Calgary

**Figure 6:**
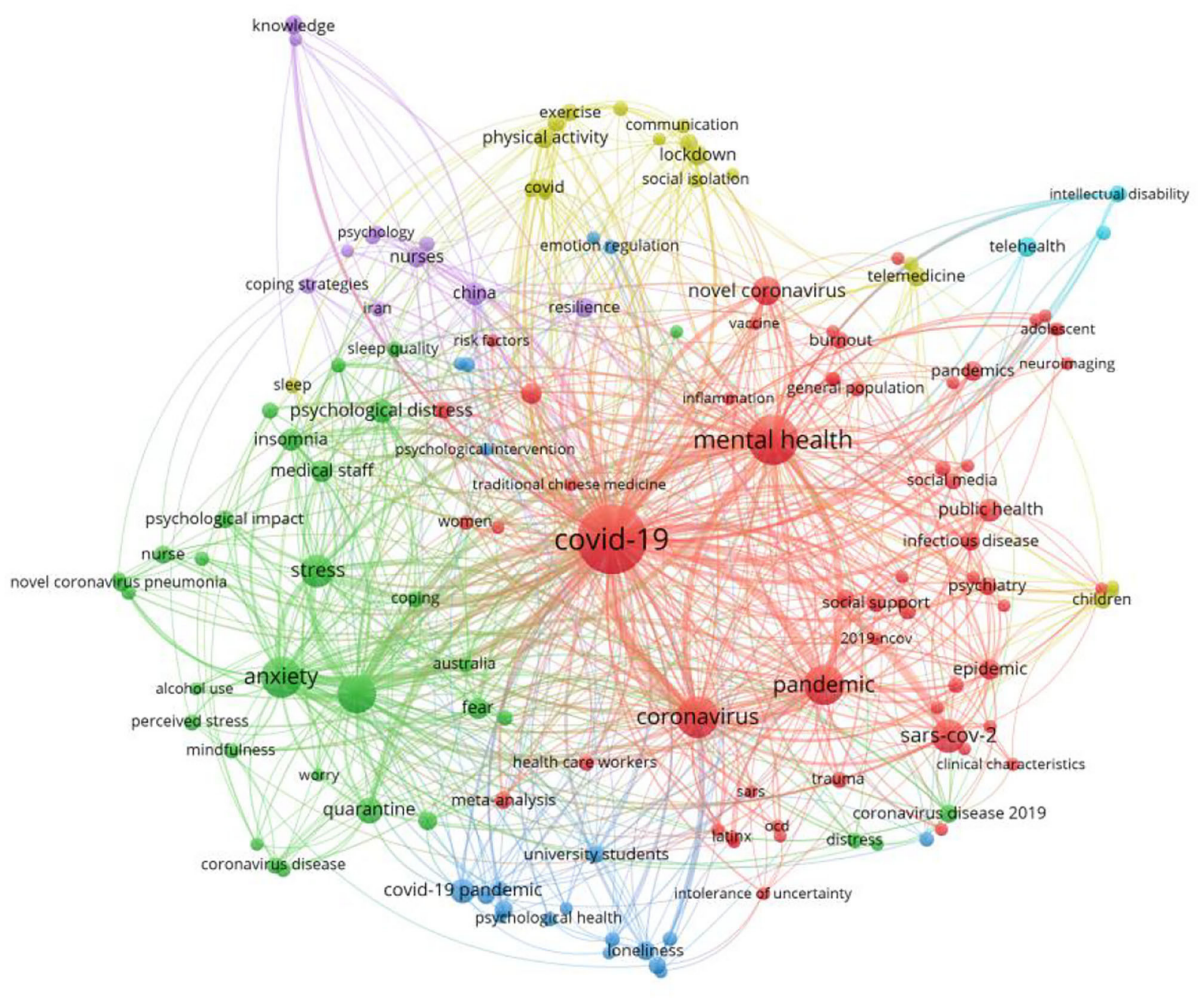
Keyword clustering.

As can be seen in Figure 6, Huazhong University of Science and Technology has led Chinese universities and research institutions, such as Shanghai Jiao Tong University and Peking University, in conducting research on COVID-19 and mental health. Hong Kong Polytechnic University, Fudan University, and the University of Melbourne acted as bridges, connecting famous universities and research institutions in Europe, America, and other countries in the world, such as Kings College London and Harvard Medical School, to jointly study issues in this field. In particular, they conduct joint research, directly or indirectly, through Hong Kong Polytechnic University, which display the important communication and joint role of Hong Kong Polytechnic University.

Judging from Table 4, the most mentioned keywords, in addition to COVID-19 and mental health, can be roughly divided into three categories: (1) keywords representing specific groups of people, such as adolescents, young adults, doctors, nurses, medical staff, and healthcare workers; (2) keywords describing influences and symptoms, such as isolation, loneliness, anxiety, depression, stress, and insomnia; and (3) keywords related to public health policies, such as lockdown, social distancing, telehealth, telemedicine, and quarantine.

**Table 4:** Keyword clustering I.

**Count**	**Centrality**	**Keyword**	**Year**	**Cluster**
227	0.54	Mental health	2020	0
16	0.1	Psychological distress	2020	0
16	0.41	Fear	2020	0
14	0	Lockdown	2020	0
13	0.1	Healthcare worker	2020	0
10	0	Psychological impact	2020	0
9	0	Adolescent	2021	0
7	0.06	Social distancing	2020	0
6	0	Burnout	2021	0
4	0	Distress	2021	0
4	0	Stigma	2020	0
4	0.05	Social media	2020	0
3	0	Trauma	2020	0
3	0	COVID-19	2020	0
2	0	Spirituality	2022	0
20	0.05	Nurse	2020	1
15	0.24	Insomnia	2020	1
14	0.46	Medical staff	2020	1
11	0.05	Resilience	2020	1
8	0.1	Sleep	2021	1
5	0	Qualitative research	2021	1
5	0	Coping	2021	1
5	0.1	Coping strategy	2021	1
4	0.15	Perceived stress	2021	1
4	0	Prevalence	2021	1
4	0	Physician	2021	1
13	0.16	Telehealth	2020	2
10	0.17	Children	2021	2
10	0.27	Telemedicine	2020	2
8	0.21	Mental health service	2020	2
7	0	Quality of life	2021	2
6	0	COVID	2020	2
6	0	College student	2021	2
5	0.21	Coronavirus disease 2019	2020	2
4	0.05	COVID19	2020	2
3	0	Viral infection	2020	2
31	0.21	Novel coronavirus	2020	3
18	0.41	Public health	2020	3
9	0.03	Infectious disease	2020	3
8	0.12	Mentalhealth	2020	3
7	0.07	Psychiatry	2020	3
7	0	Pandemics	2020	3
3	0.03	Young adult	2020	3
3	0	Risk communication	2020	3
3	0	COVID-19 outbreak	2020	3
3	0.12	Psychotherapy	2020	3
112	0.95	Coronavirus	2020	4
14	0.22	Physical activity	2020	4
9	0	Meta-analysis	2020	4
7	0.05	University student	2021	4
6	0.23	Exercise	2021	4
5	0.15	Health	2021	4
4	0	Depressive symptom	2021	4
4	0	Attitude	2021	4
3	0.05	Health care worker	2020	4
537	1.08	COVID-19	2020	5
98	0.6	Pandemic	2020	5
19	0.15	China	2020	5
13	0.66	Epidemic	2020	5
11	0	Social support	2020	5
4	0	Knowledge	2020	5
3	0.05	Psychological stress	2020	5
3	0	Psychological intervention	2020	5
2	0.19	Qualitative study	2022	5
106	0.72	Anxiety	2020	6
95	0.66	Depression	2020	6
57	0	SARS-CoV-2	2020	6
54	0.61	Stress	2020	6
10	0	Ptsd	2021	6
6	0	Outbreak	2020	6
4	0	Sleep quality	2020	6
3	0.1	Isolation	2020	6
25	0	Quarantine	2020	7
21	0.1	COVID-19 pandemic	2020	7
13	0.78	Loneliness	2021	7
10	0	Wellbeing	2021	7
7	0.78	Worry	2021	7
2	0.2	Youth	2022	7
2	0	Suicidal ideation	2022	7
2	0.34	Longitudinal	2022	7

In Graph 7, we can judge that COVID-19, mental health, pandemic, and coronavirus are represented by larger red dots as their centrality indexes are naturally higher. In this bibliometric network map, other keywords emerged next to them and together formed this visualization bibliometric network. Occupational and sociodemographic characteristics are clustered together, while symptoms of mental health problems are clustered next to them. Specific groups of people and their typical symptoms and causes occupy certain areas on the map. For example, typical symptoms of university students and the possible causes of these symptoms are grouped together on the map. Similarly, quarantine policy and its influence are also classified in certain areas. In addition, research methods and solutions appeared sporadically on this map.

Table 5 shows eight groups of core keywords separated from keyword clustering I. Each of these groups contains three keywords, which proves that these keywords appear at the same time in a considerable part of the research, and are more closely related. Keyword ClusteringII cannot only present the outline of existing mental health research in academia, but also highlights the focus of research. In addition, SiteSpaceII and VOSviewers also gave us some clues about the research trends and further development.

**Table 5:** Keyword clustering II.

**Cluster ID**	**Size**	**Silhouette**	**Mean year**	**Keyword**		
0	13	0.918	2020	Quarantine	COVID-19 pandemic	Psychological distress
1	10	0.936	2020	Epidemic	Telehealth	Telemedicine
2	10	0.925	2020	Nurse	Insomnia	Medical staff
3	9	0.737	2020	Coronavirus	Lockdown	Physical activity
4	9	0.863	2020	COVID-19	Mental health	Pandemic
5	8	0.949	2020	Novel coronavirus	Public health	Mental health
6	7	0.827	2020	Anxiety	Depression	Stress
7	6	0.887	2021	Loneliness	Health	University student

## Discussion

### Research Focuses

#### Medical Staff

The COVID-19 pandemic has exacerbated mental health problems among populations, especially medical staff, patients with COVID-19, chronic disease patients, and isolated people. Doctors, nurses, and other medical staff have significantly higher rates of insomnia than other populations (1). The researchers obtained the relevant demographic data through the WeChat questionnaire survey. Questions in the questionnaire are related to insomnia, depression, anxiety, and stress-related symptoms during the pandemic. Their research found that, since the outbreak, more than one-third of the medical staff suffered from symptoms of insomnia. Psychological intervention measures were necessary for those people (2). Research within medical institutions shows that the psychological pressure of medical staff in isolation wards was greater, but had also attracted greater attention from hospital administrators. The concern of hospital managers alleviated the pressure of medical staff to a certain extent. Further, concern for the public also reduced their psychological burden. In terms of anxiety about infection and fatigue factors, the research results showed that the psychological burden of nurses was heavier than that of doctors. Healthcare workers who lived with their own children showed more obvious fatigue and anxiety, which might be due to the fear of their children becoming infected. In terms of workload and work motivation, medical staff who have been working for more than 20 years have a heavier workload, but they can still maintain their enthusiasm to fight against the pandemic (3). Another survey showed that 73.4% of healthcare workers, mainly physicians, nurses, and auxiliary staff, reported post-traumatic stress symptoms during outbreaks, with symptoms persisting for up to 3 years in 10–40% of the cases. Depressive symptoms were reported in 27.5–50.7%, insomnia symptoms in 34–36.1%, and severe anxiety symptoms in 45% (4). A subgroup analysis revealed gender and occupational differences, with female health care practitioners and nurses exhibiting higher rates of affective symptoms compared to men and medical staff, respectively (5).

#### Quarantine

As a result, depressive symptoms (21%) and anxiety symptoms (19%) are higher during the COVID-19 pandemic compared to previous epidemiological data. About 16% of the subjects suffered from severe clinical insomnia during the lockdown. The pandemic and lockdown seemed to be particularly stressful for younger adults who were under 35 years old, women, people out of work, or those with low incomes (6). In the fight against the pandemic, China adopted measures to restrict population aggregation, such as the blockade of pandemic areas, individual patient isolation, and restrictions on the movement of people in non-pandemic areas. These measures effectively prevented the spread of the pandemic. At the same time, the use of health codes, grid-like community management, and the operational efficiency of infectious disease information networks have greatly improved. However, quarantine has also brought with it a number of problems, such as increasing psychological pressure on the population, affecting the daily lives of families, and hindering social and economic development (7). A large sample size study with wide coverage published in 2021 showed that young people quarantined at home in different provinces had different rates of anxiety and depression due to different severity of pandemic situations in different regions. The risk of anxiety and depression was statistically significantly higher in girls than in boys. The rate of anxiety and depression was affected by factors, such as gender, age, and area, as well as the existence of COVID-19 cases in the surrounding area (8).

#### Psychological Symptoms

The impact of the aforementioned isolation measures on mental health is only part of the impact of the COVID-19 on mental health. Psychological symptoms brought about by the pandemic have also been systematically sorted out by scholars. These studies show two clues. First, certain people have special psychological symptoms; second, psychological symptoms in different countries of the world are roughly the same. Several factors were associated with a higher risk of psychiatric symptoms or low psychological wellbeing, including female gender and poor self-related health (9). Relatively, severe symptoms of anxiety, depression, post-traumatic stress disorder, psychological distress, and stress were reported in the general population during the COVID-19 pandemic in China, Spain, Italy, Iran, the USA, Turkey, Nepal, and Denmark. Risk factors associated with measures of distress include female gender, younger age group, the presence of chronic or psychiatric illnesses, unemployment, student status, and frequent exposure to social media or news concerning COVID-19. The pandemic is associated with significant levels of psychological distress that, in many cases, will meet the threshold for clinical relevance. Mitigating the hazardous effects of COVID-19 on mental health is an international public health priority (1). Infectious disease pandemics often cause some people to act irrationally. The results of a survey based on psychological symptoms and irrational behaviors have drawn some conclusions. First, the vast majority of people remain in good physical and mental health, but some exhibit irrational behaviors. Second, women, elderly people, and those with confirmed cases showed more physical and mental symptoms and irrational behaviors. Finally, paradoxically, people with high education levels showed more mental symptoms, but fewer irrational behaviors (10).

#### Telemedicine

Just as the pandemic has enabled the rapid development of online education, the prospects of telemedicine are also favored by experts, observers, and investors. However, there are two restrictive aspects, namely, telemedicine equipment and telemedicine human resources. The application of 5G communication technology, telemedicine equipment, remote monitoring equipment, remote physical sign monitoring equipment, and medical artificial intelligence triage equipment all need to be urgently developed and improved. Jiangsu, a province in China, is a model province of the national project called “Internet + Medical and Health.” During the pandemic, the telemedicine by public hospitals in Jiangsu Province helped improve the efficiency of diagnosis and treatment, alleviating the pressure of offline diagnosis and treatment, and reducing the risk of cross-infection. Subsequently, medical staff were fully supportive of telemedicine. However, there was a shortage of medical staff in fever clinics, obstetrics and gynecology, pediatrics, and psychiatrists that provided telemedicine services, and they lacked corresponding incentive mechanisms (11). Effective mitigation strategies to improve mental health were developed by public health management experts. To control the rapid spread of COVID-19 and manage the crisis better, both developed and developing countries have been improving the efficiency of their health system by replacing a proportion of face-to-face clinical encounters with telemedicine solutions (12).

#### Social Media

There were rumors in various kinds of media during the COVID-19 pandemic. Although we can regard rumors as a disturbing error for psychological measurement, if they are not strictly controlled, their impact on people's mental health and behavior cannot be ignored. A study focusing on the spread of WeChat rumors has explored the psychological perception mechanism of audiences affected by rumor spreading in emergency situations. The study has significant results in the following terms: the form characteristics of the rumors in COVID-19, the ranking of susceptible age groups, the degree of dependence of the test subject on certain media and its psychological impact, and the follow-up behavior of the test subjects related to psychological variables (2). In 2021, another interesting study based on the data of TikTok videos released by three mainstream media in China showed that they inevitably caused some psychological trauma to the public. However, from the perspective of overall emotional orientation, short-format videos with positive reporting emotional tendencies had an advantage in attracting likes from TikTok users. Positive government responses to pandemic information were very important, and those responses could be recognized and praised by most social media users. Some of the TikTok videos, such as The Plasma of a Recovered Patient Cured 11 Other ICU Patients, The First COVID-19 Test Kit Passed Inspection, and A Frenchman Named Fred gave up Returning to Home to Join China's Anti-COVID-19 Battle, are extremely popular among social media users. Most social media users have been providing spiritual sustenance for people in the pandemic (13). When a public health crisis occurs, social media plays an important role in increasing public vigilance, helping the public identify rumors, and boosting public morale.

#### University Students and Loneliness

A study that assessed the adverse impact on the mental health of university students has drawn some conclusions. First, the severity of the outbreak has an indirect effect on negative emotions by affecting sleep quality. Second, a possible mitigation strategy to improve mental health includes ensuring suitable amounts of daily physical activity and deep sleep. Third, the pandemic has reduced people's aggressiveness, probably by making people realize the fragility and preciousness of life (14). Another research focused on social networks and mental health compared two cohorts of Swiss undergraduate students who were experiencing the crisis, and made an additional comparison with an earlier cohort who did not experience the pandemic. The researchers found that interaction and co-study networks had become sparser, and more students were studying alone. Stressors shifted from fear of missing out on social life to concern about health, family, friends, and their future (15). Young adults, women, people with lower education or lower income, the economically inactive, people living alone, and urban residents were at greater risk of being lonely during the pandemic. Being a student emerged as a higher than usual risk factor for loneliness during the lockdown (16). A study to explore the relationship between loneliness and stress among undergraduates in North America showed that the loneliness and stress among college students increased. On one hand, stress plays a key role in the deterioration of college students' mental health; on the other hand, reducing the loneliness of college students is expected to reduce the negative impact of stress on college students' mental health (17).

### Research Trends

Due to the limited training sample of academic papers at present, it is difficult to predict the outcomes accurately. Though we cannot exactly predict the hot issues in the future, we can sort out some possible research trends in this field by analyzing existing research approaches. Psychological symptoms that affected people's mental health during the COVID-19 pandemic will be discovered further, especially those that probably continued to affect people's mental health even after the pandemic is controlled.

Studies on mild psychological symptoms, such as mild insomnia and anxiety, tend to decrease slowly, and in the case of severe problems caused by the pandemic, or severe psychological symptoms, such as clinical insomnia, depression, bipolar disorder, the corresponding in-depth research will continue. The impact of a global pandemic on the mental health of the global population must be profound and worthy of study. Due to the rapid development of COVID-19, many famous universities and research institutions have not had enough time to collect sufficient data and relevant research materials. The different effects on populations in different countries with different pandemic prevention policies are not yet fully displayed.

Regardless of how research on mental health develops, the COVID-19 pandemic has indeed brought us some new insights. As mentioned in many articles on mental health interventions for adolescents and college students, the mental health of specific populations and the development of telemedicine all deserve continued academic attention. Mental health intervention for adolescents and college students is a means to consider and prepare for the future. To ensure responsible and accountable behavior for future generations, we should all pay attention to the research and application of this method. Caring for specific groups of people, such as doctors, nurses, and other healthcare workers, and studying how to protect them in a global pandemic is a topic that global academia must study in the future, or we will lose protection the next time the virus sweeps the world. In addition, telemedicine is the trend in the future, and face-to-face diagnosis and treatment will undoubtedly increase the risk of cross-infection during the pandemic. Therefore, the development of telemedicine is an important way to avoid contact between the patients. The COVID-19 pandemic has accelerated the research and development of telemedicine.

### Limitations

(1) Though we have selected three databases for analysis, there are still some databases that may be related to this field that are not covered in this study. (2) Since COVID-19-related research was started just 2 years ago, the results of the bibliometric analysis may vary after adding new data. (3) The citation frequency of articles is influenced by the time of publication, thus previously published articles should be cited more frequently than new ones. (4) Bibliometric data change over time, and different conclusions may be drawn over time. Therefore, this study should be updated in the future.

## Conclusions

The most mentioned keywords, in addition to COVID-19 and mental health, can be roughly divided into three categories: keywords representing specific groups of people, keywords describing influences and symptoms, and keywords related to public health policies. The most mentioned issues were about medical staff, quarantine, psychological symptoms, telemedicine, social media, and loneliness. Mild psychological symptoms, such as insomnia, depression, and anxiety, tend to decrease slowly, while severe ones, such as severe clinical insomnia, depression, and bipolar disorder, are yet to be discovered. The importance of studies on the protection of youth medical staff and telemedicine studies will become even more significant in the future. While physical health is threatened by the pandemic, human mental health also suffers. Judging from the current situation of pandemic prevention and control, if severe prevention and control measures are taken, the impact of COVID-19 on the health of the social population is controllable; if a strategy of coexistence with the virus is adopted, as long as a new deadly mutation of COVID-19 does not emerge, the outcomes can be controllable. However, the impact of the pandemic on human mental health is not easy to predict. In addition to the abovementioned papers on mental health, the author also noted that some papers focused on neuromedicine pointed out that the virus might have some damage to the normal working mechanism of the human nervous system, but these studies are outside the scope of mental health research, at least for now. This study aims to summarize the observations, analysis, and research of scholars on mental health during the pandemic from 2020 to early 2022, with a view to provide more clues for future researchers. We hope that more researchers will build on our research to discover new research areas and new questions to help more countries, groups, and individuals affected by the COVID-19 pandemic.

## Data Availability Statement

The raw data supporting the conclusions of this article will be made available by the authors, without undue reservation.

## Author Contributions

YL was responsible for the concept and design, drafting this article, and bibliometric analysis. YL, LS, and XT were responsible for the revision and data collection. All authors contributed to this article and approved the submitted version.

## Conflict of Interest

The authors declare that the research was conducted in the absence of any commercial or financial relationships that could be construed as a potential conflict of interest.

## Publisher's Note

All claims expressed in this article are solely those of the authors and do not necessarily represent those of their affiliated organizations, or those of the publisher, the editors and the reviewers. Any product that may be evaluated in this article, or claim that may be made by its manufacturer, is not guaranteed or endorsed by the publisher.
